# Cytoskeletal alteration modulates cancer cell invasion through RhoA-YAP signaling in stromal fibroblasts

**DOI:** 10.1371/journal.pone.0214553

**Published:** 2019-03-28

**Authors:** Do Kyeong Kim, Eun Kyoung Kim, Da-Woon Jung, Jin Kim

**Affiliations:** 1 Oral Cancer Research Institute, Department of Oral Pathology, BK21 PLUS Project, Yonsei University College of Dentistry, Seoul, Republic of Korea; 2 Department of Dental Hygiene, Jeonju Kijeon College, Jeonju, Republic of Korea; 3 New Drug Targets Laboratory, School of Life Sciences, Gwangju Institute of Science and Technology, 1 Oryong-dong, Gwangju, Republic of Korea; Chung Shan Medical University, TAIWAN

## Abstract

Cancer-associated fibroblasts(CAFs) participate in carcinogenesis through interaction with cancer cells. This study aimed to investigate the mechanism of cytoskeletal alteration of CAFs and its role in invasion of oral squamous cell carcinoma(OSCC).Immortalized normal fibroblasts(hTERT-hNOFs) co-cultured with OSCC cells showed myofibroblastic and senescent phenotypes like CAFs. Thus, this study substituted hTERT-hNOFs for CAFs. Next, the cytoskeletal alteration and its molecular mechanism were investigated in hTERT-hNOFs co-cultured with OSCC. As results, we found that RhoA regulated cytoskeletal organization in fibroblasts surrounding OSCC cells. Furthermore, as a downstream transcriptional factor of RhoA, YAP was mainly localized in the nucleus of hTERT-hNOFs co-cultured with OSCC. Consequently, we examined whether nuclear YAP localization of fibroblasts could influence cancer progression. YAPS127A fibroblasts manifesting nuclear localization of YAP induced cytoskeletal alteration and increased gel contractility and matrix stiffness, and thereby enhances the invasiveness of OSCC cells. In conclusion, the modification of tumor microenvironment, such as cytoskeletal change and matrix remodeling *via* RhoA-YAP in CAFs, modulates OSCC invasion. These understandings will provide the development of novel approaches for CAFs-based cancer therapy.

## Introduction

Carcinomas are not only composed of malignant epithelial cells, but also organized as complex with tumor microenvironment through reciprocal interaction[1]. In the tumor microenvironment, Cancer-associated fibroblasts (CAFs) are the most abundant cells that can participate in carcinogenesis[1,2], including angiogenesis[3] and metastasis[4,5].

CAFs are characterized by myofibroblastic- and senescent phenotypes[2,6,7]. The myofibroblastic phenotypical CAFs heterogeneously expressed the markers of activated fibroblasts, including α-smooth muscle actin (α-SMA), fibroblast-activation protein(FAP) and fibroblast-specific protein 1 (FSP1)[2,8]. As the characteristics of senescent fibroblasts, IL-6 and IL-8, senescence associated secretory protein (SASP),are released from CAFs[9]. Also, oncoproteins, including Caveolin-1[10,11], CSL and p53[12] could maintain the state of senescent CAFs. Most cancer therapies have mostly focused on neoplastic epithelial cells, while little attention to CAFs.

Unlike tumor cells, CAFs are genetically stable. With these reasons, CAFs have been emerged to be an attractive therapeutic target for reducing risk of drug resistance and tumor recurrence[13–16]. Several molecules have already been suggested as promising targets of CAFs-based therapy; Targeting FAP, SDF-CXCR4, HGF-Met, TGF-β, PDGF-C and VEGF signaling pathways attenuated tumor growth and tumor progression, as well as reducing innate drug resistance[17–19]. The drugs to CAF-based therapy can partially block the function of CAFs and some of them are even in clinical trials, such as Sibrotuzumab[20] or Talabostate[21] targeting FAP and other drugs targeting TGF-β signaling[22,23]. However, there are still many limitations in clinical trial; well-known CAF markers, α-SMA and FAPα, are often without distinction from normal fibroblasts or even higher expression in normal fibroblasts[8]. In addition, these CAFs markers are detected in other non-cancerous cell type, such as pericytes and smooth muscle cells or mesodermal cells[24]. Thus, it is not enough to target CAFs specifically in clinical trials yet. Importantly, the promising molecule for CAF-based cancer therapy should be developed to improve their specificities and efficacies.

CAFs exhibited cytoskeletal changes, including forced contractility and tissues stiffness, and then increased proteolytic activity to remodel the extracellular matrix (ECM) adjacent to cancer[25,26]. Despite of the association of tissue mechanical features in cancer, the mechanical role of CAFs remains poorly understood in cancer progression. Therefore, this study aimed to investigate the cytoskeletal alteration and its molecular mechanism of CAFs, and further to examine its role in invasiveness. This study will contribute to develop novel approaches for CAFs-based cancer therapy.

## Materials and methods

### Reagents and antibodies

Antibodies for α-SMA (1:100, mouse monoclonal, Dako, Glstrup, Denmark), Vimentin (1:1000, rabbit monoclonal, Cell signaling technology, Beverly, MA, USA), Phospho-FAK (Tyr397) (1:1000 Cell signaling technology, Beverly, MA, USA), FAK (1:1000, rabbit monoclonal, Cell signaling technology, Beverly, MA, USA), YAP (1:200, mouse monoclonal, Santa Cruz, CA, USA), phospho-YAP (1:1000, rabbit monoclonal, Cell signaling technology, Beverly, MA, USA), Lamin B1 (1:1000, rabbit monoclonal, Cell signaling technology, Beverly, MA, USA), β-tubulin (1:1000, Cell signaling technology, Beverly, MA, USA) and β-actin (1:5000, Bioworld Technology, St. Louis, MN) were used, respectively. Horseradish peroxidase-conjugated anti-mouse or anti-rabbit secondary antibodies were obtained from Cell Signaling Technology (Beverly, MA, USA). The pharmacological inhibitors, Y27632 and Latrunculin A (Lat.A) were obtained from Enzo Life Sciences (Farmingdale, NY), were used to block RhoA downstream kinase (ROCK) and actin polymerization, which can prevent the F-actin assembly by forming the complex between Lat.A and G-actin. For invasion assay, type I collagen (Cellmatrix type 1-A) was purchased from Nitta Gelatin (Osaka, Japan). For ELISA immunoassay, recombinant human IL-6 and IL-8 (standard), human/Primate IL-6 (2μg/ml) / IL-8 (8μg/ml) Antibody (Capture) and Human/Primate IL-6 (2.5μg/ml) / IL-8 (1μg/ml) Biotinylated Antibody (Detection) were obtained from R&D systems. For immunofluorescence, Phalloidin-Tetramethylrhodamine B isothiocyanate and diamidinophenylindole (DAPI) were purchased from Sigma Aldrich (MO, USA). The cells cover-slipped with Dako fluorescent mounting medium (Dako, CA, USA)

### Cell culture

The immortalized human gingival fibroblasts by hTERT-transfection (hTERT-hNOFs) were used, which is previously described[27,28]. CAFs, which were obtained from the surgical specimens of OSCC patients, and NOFs, which were derived from three patients without mucosal disease who underwent wisdom tooth extraction, were used for conducting the study. CAFs and NOFs were primary cultured in Yonsei University College of Dentistry (IRB 2-2012-0027)[19]. All fibroblasts were then maintained in F medium which is composed of Dulbecco’s modified Eagles medium (Gibco BRL, NY, USA) and F-12 Ham (Ham’s F12; Gibco BRL, NY, USA) mixed in a 3:1 ratio, and supplemented with 10% fetal bovine serum and 1% penicillin/streptomycin. Normal human epidermal keratinocytes (HEK) were also used for this study and maintained in keratinocyte growth media with supplementary bullet kit (KGM; Lonza, Walkersville, MD, USA)[28]. YD10B and YD38 OSCC cells were obtained from tongue and lower gingiva of OSCC patients, respectively [29]. YD OSCC cells were maintained in EF medium (the mixture of F medium and E medium at 9: 1 ratio). E medium is composed of 0.01 μg/ml cholera toxin, 0.04 μg/ml hydrocortisone, 0.5 μg/ml insulin, 0.5 ug/ml apo-transferrin, and 0.2 μg/ml 3´-5-triodo-1-thyroine (Sigma, MO, USA) in F medium. All cells were maintained at 37°C in an incubator containing 5% CO_2_. The authentication of all cell lines was verified by STR DNA profiling in Korean Cell Line Bank.

### RNA extraction and RT-PCR

Total RNA was extracted from each cells using RNeasy plus mini kit (Qiagen, Hilden, Germany), and cDNA was synthesized from 1 μg of the total RNA by using RT&GO-MasterMix (MP Biomedicals, CA, USA) according to the manufacturers’ protocols. The primer sequences for PCR are shown as following. IL-6; forward(F): 5’-ATGAACTCCTTCTCCACAAG-3’, reverse(R): 5’-GAAGAGCCCTCAGGCTGGAC-3’, IL-8; F: 5’-AGACAGCAGAGCACACAAGC-3’, R: 5’-TTGGGGTGGAAAGGTTTGGAG-3’, FAK; F: 5’-TTTCGACGTTTTACCTCAGC-3’, R: 5’-AGGGTAGGAGGACAATTTGG-3’,RhoA; F: 5’-TCTTCAGCAAGGACCAGTTC-3’, R: 5’-CCAACTCTACCTGCTTTCCA-3’,Rac1; F: 5’- GAAGAAGCTGACTCCCATCA -3’, R: 5’- ACAACAGCAGGCATTTTCTC -3’, Cdc42; F: 5’- CAGATTACGACCGCTGAGTT -3’, R: 5’- GAGTCCCAACAAGCAAGAAA -3’,YAP; F: 5’- GCTACAGTGTCCCTCGAACC -3’, R: 5’-CCGGTGCATGTGTCTCCTTA-3’,CTGF; F: 5’-ATCTTCGGTGGTACGGTGTA-3’, R: 5’-ACGTGTCTTCCAGTCGGTAA-3’,GAPDH; F: 5’-GAAGGTGAAGGTCGGAGT-3’, R: 5’-GAAGATGGTGATGGGATTTC-3’. The reaction mixture was subjected to 30 RT-PCR amplification cycles 40 s at 94°C, 58 s at 48°C and 40 s at 72°C and the products were loaded in 1–1.5% agarose gel using StaySafe Nucleic Acid Gel Stain (Real Biotech Corporation, Taipei, Taiwan). The results of RT-PCR were normalized to GAPDH.

### Enzyme linked immunosorbent assay (ELISA)

For collecting co-culturing conditioned medium, All fibroblasts (2 × 10^5^) were grown in the lower chamber and then OSCC cells (2 × 10^5^) were seeded in 6-transwell plates containing collagen-coated 0.4 μm pore transmembrane filters (Corning-costar, Lowell, MA, USA) for 48 h. In case of mono-culturing medium, only fibroblasts were seeded in lower chamber of 6-well plate. The mediums were collected by centrifugation, and protein concentration was estimated using Pierce BCA protein assay kit (Thermo Fisher Scientific). In briefly, capture antibodies were attached in microplates at overnight. Then, conditioned mediums and standard recombinant proteins were added and bind to capture antibodies for 2 h. After washing steps, the antibodies for detection were incubated for 1 h. After removal of excess detection antibodies, an HRP conjugate streptavidin was added and subsequently, TMB solution and H_2_SO_4_ solution was added. The intensity of colored product was analyzed by M680 microplate reader (Bio-Rad). All results were normalized for total protein concentration and indicated as pg/mg of protein.

### Protein extraction and Western blotting

When fibroblasts co cultured with OSCC cells, the fibroblasts (2.5 × 10^5^) were seeded in lower chamber and then co cultured with OSCC cells or HEK cells (2.5 × 10^5^) of upper chamber in 6-well plates. In brief, cell was lysated by cell lysis buffer (Cell signaling technology, Beverly, MA, USA) and then harvested. After lysing, protein lysates were incubated for 30 min on ice with vortexing every 5 min. The lysates were centrifuged at maximum speed (>15,000 rpm) for 10 min. Subsequently, the lysates were boiled for 5 min in sodium dodecyl sulfate (SDS) sample buffer and separated on 10% SDS-PAGE. For fraction of nuclear cytoplasmic protein, NE-PER Nuclear and Cytoplasmic extraction reagents (Thermo Fisher Scientific Inc. USA) were used, respectively, according to instructions. The proteins were transferred to polyvinylidene difluoride(PVDF) membrane, blocked in 5% milk in Phosphate Buffered Saline with Tween 20 (PBST). The membranes were immunoblotted with appropriate primary antibodies and then incubated with horseradish peroxidase-conjugated anti-mouse or anti-rabbit secondary antibodies (Cell signaling technology, Beverly, MA, USA) and detection was done by chemiluminescence (GenDEPOT, TX, USA).

### Senescence-associated β-galactosidase(SA-β-Gal) staining

In co-culturing condition, hTERT-hNOFs (1.5 × 10^5^) were seeded and incubated overnight in a 6-well lower chamber and OSCC cells (1.5 × 10^5^) or HEK cells (1.5 × 10^5^) were added in 6-well upper chamber, respectively. In mono-culturing condition, only fibroblasts were grown in 6-well plates. After indicated times, cells were stained using β-galactosidase staining kit (Cell signaling technology, Beverly, MA, USA) and incubated overnight at 37°C without maintaining 5% CO_2_. The cells were washed with PBS and 70% glycerol was added for storage. Stained cells were identified and counted by light microscopy (Olympus, Tokyo, Japan).

### Immunofluorescence

In co-culturing condition, sterilized cover glasses (20 x 20 mm, Deckglaser) were put into 6-transwell plates containing collagen-coated 0.4 μm pore transmembrane filters (Corning-costar, Lowell, MA, USA). Then, fibroblasts were seeded onto cover glasses in lower chamber and OSCC cells were incubated in upper chamber for indicated times. In CAFs and NOFs, the fibroblasts were seeded into chamber slide (Lab-Tek Chamber slide, Nalge Nunc, Roskilde, Denmark). The cells were stained with appreciated antibodies (Phallodin(F-actin), YAP) following manufacturer’s instructions. The nuclei were stained with 10 μg/ml DAPI and then Dako fluorescent mounting medium was used to cover-slipped.

### RhoA-GTP pull down assay

For measurement of RhoA activiation, RhoA activation assay Kit (abcam, Cambridge, UK) was utilized by manufacturer’s instructions. In brief, the fibroblasts were harvested by 1X Assay/Lysis Buffer and then the lysates centrifugation for 10 min (12,000 x*g* at 4°C). The lysates were added anti-Active RhoA mouse monoclonal antibody and subsequently incubated by gently agitating with the protein A/G agarose bead slurry for 1 h. After that, the samples were centrifugated for 1 min at 5,000 *g*. After washing step, 2X SDS sample buffer was added in the bead pellet. Then, proteins were transferred to PVDF membrane. GTP gamma S was used as a positive control and GDP was used as a negative control.

### Plasmids and transfection

pcDNA4/HisMaxB mammalian expression vector was purchased from Invitrogen (#V864-20(43-0078F), USA). pcDNA4/HisMaxB-YAP1 (#18978) and pcDNA4/HisMaxB-YAP-S127A (#18988) were obtained from Addgene (Cambridge, MA, UK). In brief, prepared plasmids were amplified using Plasmid Plus Midi Kit (Qiagen, Hilden, Germany) and then selected by ampicillin (Sigma, MO, USA). Purified plasmid DNA was transfected using 4D-nucleofector X Kit for mammalian fibroblasts and Amaxa 4D-nucleofector (Lonza, Walkersville, MD, USA), following as manufacturer’s instructions. Zeocin selection reagent (#R250-01) (100 μg/ml) was used to select transfected cells. The transfection results were confirmed by DNA sequencing analysis (Bioneer, Co., Daejeon, Korea). In brief, DNA was extracted from WT-YAP-transfected and YAPS127A-transfected hTERT-hNOFs by using DNA Mini Kit (Qiagen, Hilden, Germany), and thereby RT-PCR was performed as indicated in RT-PCR method above. The following primer sequences to detect the mutation of phosphorylation site (serine at 127) in YAP; 5’- GTCATGAACCCCAAGACG -3’ (forward) and 5’- GGCAGAGGTACATCATCAGG-3’ (reverse). After electrophoresis, the exact DNA fragment was cut out with a scalpel and then DNA sequencing analysis was identified from Gel DNA extraction (iNtRON, Korea). Genomic identities were confirmed using BLASTN (http://www.ncbi.nlm.nih.gov/BLAST).

### ECM-remodeling assay

To assess the matrix remodeling mediated by fibroblasts, respectively, the fibroblasts were embedded in 500 μl of collagen Type I and seeded into. Surrounding the mixtures, F medium was added to maintain the cell. Gel contraction was monitored with the naked eye daily. After 5 days, the gel contraction value was obtained and the relative diameters of the well and the gel were measured using Image J software.

### Atomic force microscopy (AFM)

To check the elastic modulus, the fibroblasts were embedded in collagen type I. After 3 days, the edge of gels was cut with scalpel and gently moved to cover glass. The elastic modulus of the gels was measured by using Atomic force microscopy (AFM) in Yonsei Center for Research Facilities. AFM measurements were performed with a JPK Nanowizard-I (JPK instruments) interfaced to an inverted optical microscope. Pyramidal C-AFM cantilever was used with 10 μm tip sphere. Before the experiments, the sensitivity of the cantilever was set by measuring the slope of force-distance curves on empty region of glass. Using the optical microscope, the tip of the cantilever was aligned over three regions in each gel. The data represented on the average of Young’s modulus.

### Transwell migration and invasion assay

In transwell migration assay, OSCC cells (2 × 10^5^) were seeded in Transwell cell culture chamber inserts (24-transwell plates, 8 μm pore size; Costar, Cambridge, MA) in the presence of P medium. The lower chamber contained P media supplemented with 1% FBS. After 48 h of incubation, the cells on the upper surface of the filter were removed with a cotton. The cells that had migrated to the lower compartment were fixed in 10% formalin solution and stained with 0.025% crystal violet. The membrane filters were cut and mounted on slides, and then the cells were counted in five images per well (200x magnification). Each assay was performed in triplicate. Transwell invasion assays were performed in the same manner with transwell migration assay. Additionally, the inserts containing 8 μm pore size filters were coated with collagen Type I (45 μg/30 μL/well) and then solidified for overnight in 37°C incubator. After hardening the collagen, OSCC cells (2 × 10^4^) were seeded in the transwell chambers with porous filters in the upper wells. The fibroblasts in P medium containing 1% FBS was added into the lower well to induce invasive capacity of cancer cells onto upper wells. The cells that penetrated the filter were fixed, stained with 0.025% crystal violet and counted by light microscopy.

### Three-dimensional (3D) raft culture

Collagen mixture was prepared by mixing eight volumes of type I collagen solution (Nitta Gelatin, Osaka, Japan), one volume of 10× reconstitution solution (0.022 g/ml NaHCO_3_, 0.0477 g/ml HEPES and 0.05 N NaOH), and one volume of 10× P medium. The fibroblasts (hTERT-hNOFs and CAFs) (1.5 × 10^5^) were mixed with 250 μl collagen mixture. The cell-collagen mixture was placed into a Mill-cell (3.0 μm pore size, 12 mm diameter; Millipore, Billerica, MA, USA) and allowed to be polymerized at 37 ºC for 24 h. OSCC cells (YD10B and YD38) (3 × 10^5^) were seeded on top of the fibroblast-collagen mixtures. The cultures were incubated for 4 days and raised to the air-liquid interphase for 14 days. The rafts were fixed, embedded in paraffin, sectioned and stained using hematoxylin and eosin.

### Small interfering RNA(siRNA) transfection

YAP small interfering RNA(siRNA) had the following sense and antisense sequences: siYAP#1 (#1164680); Sense ‘CAGGAAUUGAGAACAAUGA’ and Antisense ‘UCAUUGUUCUCAAUUCCUG’, siYAP#2 (#1164677); Sense ‘CAGAAGAUCAAAGCUACUU’ and Antisense ‘AAGUAGCUUUGAUCUUCUG’. A negative control siRNA was used (Bioneer). Transfection of siRNA was performed using the Lipofectamine RNAiMAX (Invitrogen) according to the manufacturers’ instructions. In brief, hTERT-hNOFs (2 × 10^5^) were seeded per well in a 6-well plate and cultured for 24 h. YAP siRNAs were diluted with 250 μl Opti-MEM (Invitrogen). These two dilution mixtures were combined, mixed gently, and incubated for 20 min at room temperature to facilitate complex formation. The 500 μl siRNA-Lipofectamine mixture was then added to cells. The transfected cells were cultured for 48 h and then assayed.

### Statistical analysis

All statistical analyses were analyzed by using the SPSS version 24 (SPSS Inc., Chicago, IL, USA). Mann-Whitney U test and Student *t* test (Independent *t* test) were used to determine the statistical significant difference. All of the variables were tested in three independent experiments, and each experiment was performed at least in triplicate. The results are shown as the mean ± standard deviation (SD). The value of *p* < 0.05 was considered statistically significant.

## Results

### The immortalized fibroblasts co-cultured with OSCC cells harbor CAFs-like characteristics

**Fig 1:**
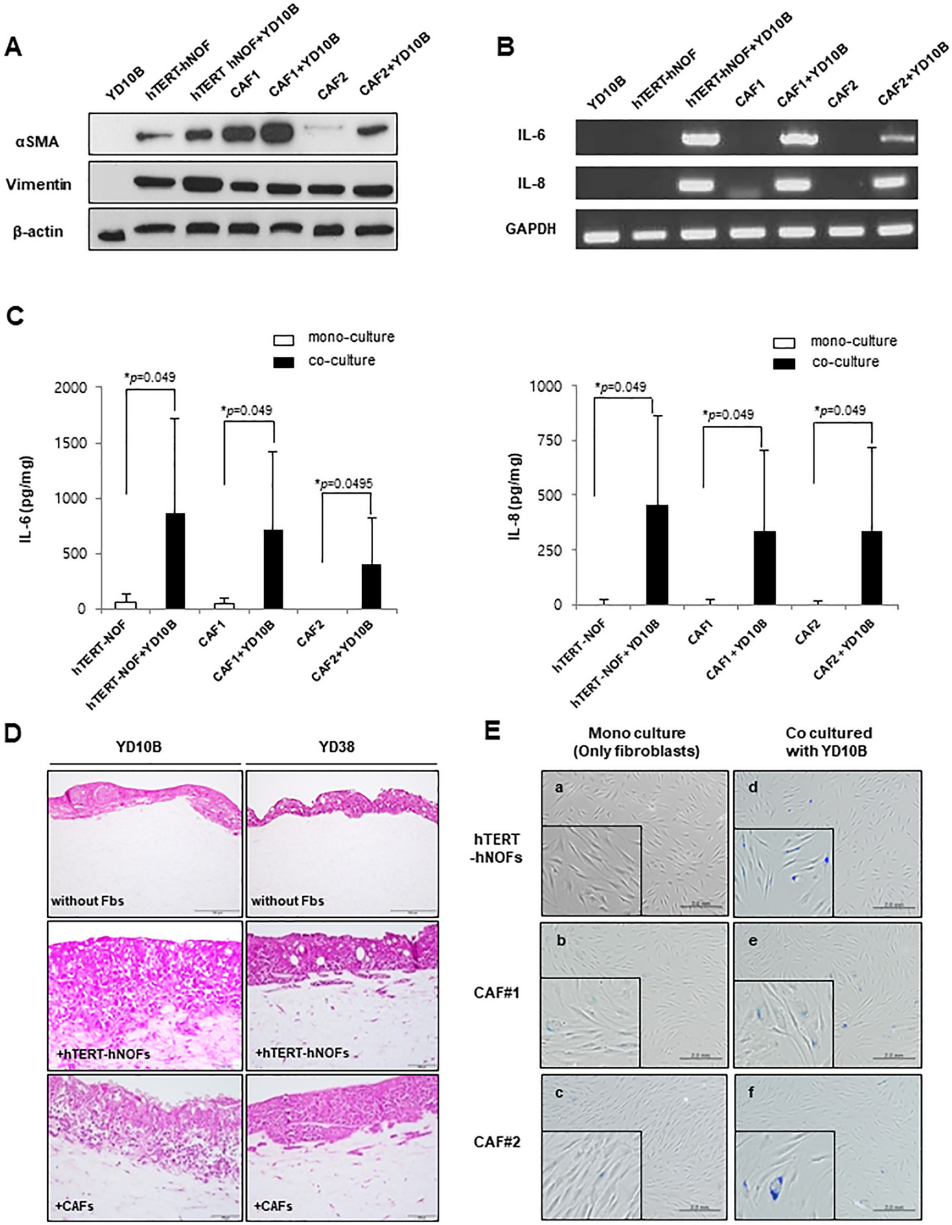
CAF like characteristics in hTERT-hNOFs by co-culturing with OSCC cells. (A) Expression of αSMA and vimentin in fibroblasts (hTERT-hNOFs and CAFs) mono-cultured and co-cultured with OSCC YD10B cells for 48 h. Cell lysates were prepared, and then Western blot were performed. (B) Cells grown in the presence or absence of YD10B OSCC cells. After 24h, the cells were harvested and detected mRNA expression of IL-6 and IL-8. (C) After 24h, conditioned medium was collected and the secretion levels of IL-6 and IL-8were measured by ELISA. The results were normalized for total protein and expressed as pg/mg of protein. This assay was performed in independent triplicate repeats (*p < 0.05; Mann-Whitney *U* test). (D) Representative images are shown to observe OSCC cells (YD10B and YD38) invaded toward stromal proportion with or without fibroblasts (hTERT-hNOFs and CAF#1) in 3D culture conditions. The mixture of each fibroblast and collagen was put into Mill-cell and grown for 14 days. Thereby, the rafts were fixed, embedded in paraffin, sectioned and stained using hematoxylin and eosin. (200x magnification, Scale bar: 100 μm, respectively). (E) SA-β-Gal assay was performed and representative microscopic pictures (a) mono-cultured hTERT-hNOFs (left, top), (d) hTERT-hNOFs, co-cultured with YD10B OSCC cells (right, top), (b) CAF#1 mono cultured (left, middle) and (e) co cultured with YD10B OSCC cells (right, middle) and (c) CAF#2 mono cultured(left, bottom) and (f) co cultured with YD10B OSCC cells (right, bottom) are shown at 72 h (magnification: 200X, scale bar: 2.0 mm). Enlarged images are shown in the bottom of each left.

As a substitution for CAFs, we used immortalized fibroblasts (hTERT-hNOFs) to overcome individual variation of primary cultured CAFs. First, we measured expression levels of myofibroblast differentiation markers, such as α-SMA and Vimentin. CAFs stimulated by OSCC cells showed increased α-SMA protein expression, even though the respective baseline expression level differed between CAF1 and CAF2 (Fig 1A). The expression and secretion of IL-6 and IL-8 were also increased in CAFs exposed by OSCC cells (Fig 1B and 1C). Consistently, when hTERT-hNOFs co-cultured with OSCC cells, α-SMA protein expressions were upregulated (Fig 1A). Also, hTERT-hNOFs co-cultured with OSCC cells secreted higher level of IL-6 and IL-8, as compared with mono-cultured cells (Fig 1B and 1C). We next investigated whether hTERT-hNOFs functionally promote the invasion of cancer cells in organotypic 3D culture models. Consistent with OSCC cells co-cultured with CAFs, OSCC cells co-cultured with hTERT-hNOFs invaded into stroma (Fig 1D).

Because CAFs show senescent features, we observed whether hTERT-hNOFs also possessed a senescent phenotype. When co-cultured with YD10B OSCC cells, SA-β-Gal positive cells was markedly increased in both CAFs and hTERT-hNOFs, compared to mono-cultured fibroblasts (Fig 1E). To support these results, the fractions of proliferating cells and senescent cells were measured in hTERT-hNOFs co-cultured with HEK or OSCC cells by double immunofluorescence staining with Ki67 and p16 (S1 Fig). Co-culturing hTERT-hNOFs with OSCC cells showed higher fraction of p16 positive cells and a decline of Ki67 positive cells, compared to those co-cultured with HEK. The senescent phenotypical change was induced by a variety of OSCC cells (S2A Fig), and was the specific feature of hTERT-hNOFs adjacent to OSCC, not to normal epithelium (S2B Fig). These results suggested that co-cultured hTERT-hNOFs with OSCC cells were biologically similar to CAFs. Based on these data, our experiments were conducted with hTERT-hNOFs.

### Cytoskeletal alteration of CAFs adjacent to OSCC cells

**Fig 2:**
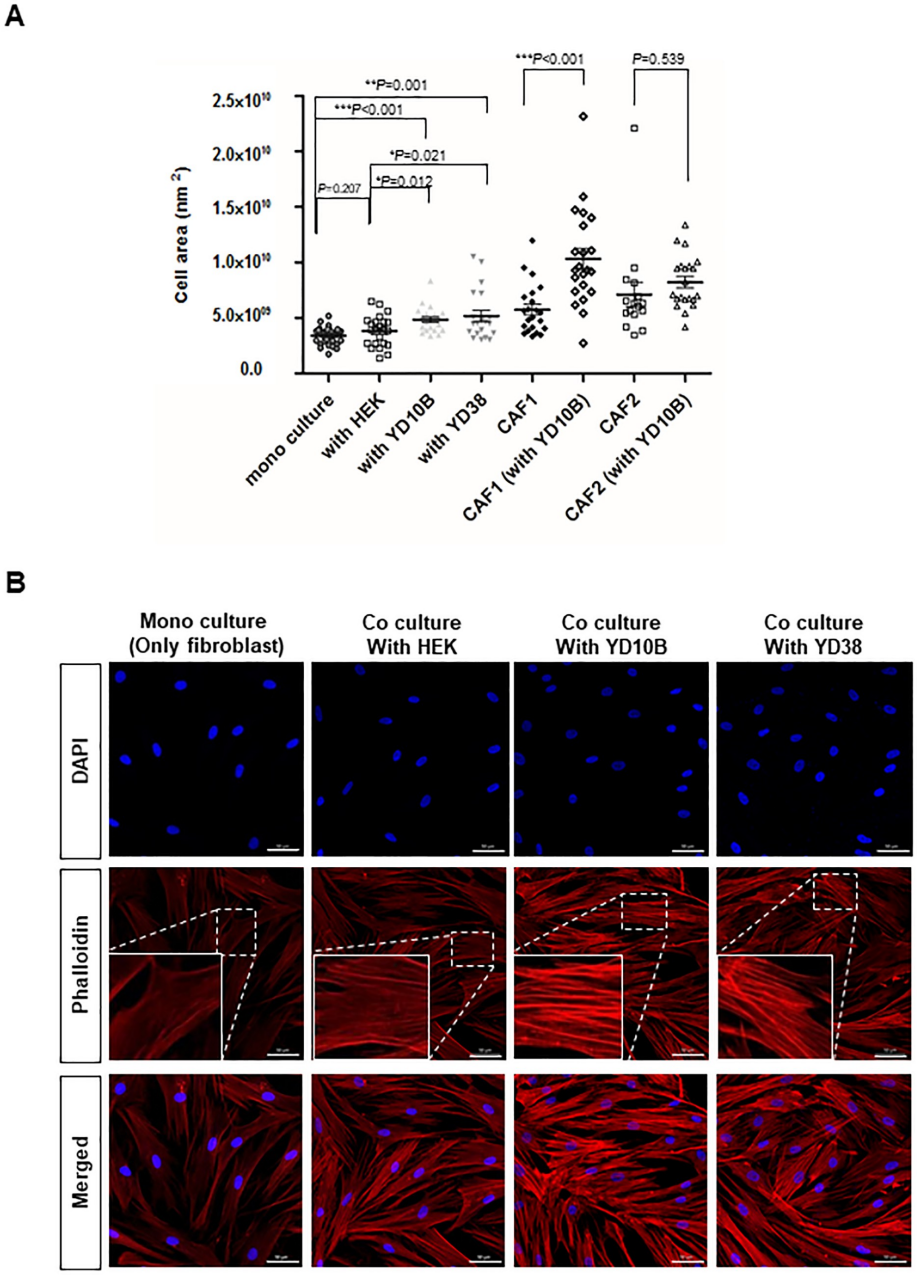
Cytoskeletal alteration in fibroblasts adjacent to OSCC cells. (A)Cell size was measured on hTERT-hNOFs mono-cultured and co-cultured with HEK and OSCC cells for 3 days. ZEN 2012 software program was used (mono cultured, n = 30; with HEK, n = 23; with YD10B, n = 21; with YD38, n = 20; CAF#1, n = 22; CAF#1(with YD10B), n = 22; CAF#2, n = 16; CAF#2(with YD10B), n = 21) (*p < 0.05, **p < 0.01, ***p < 0.001; Student *t* test). CAFs were used to compare cellular size in fibroblasts, respectively. Average of cellular size is shown as CAF#1, 5.74 x 10^9^ nm^2^; CAF#1 (with YD10B), 1.03 x 10^10^ nm^2^; CAF#2, 7.12 x 10^9^ nm^2^and CAF#2 (with YD10B), 8.22 x 10^9^ nm^2^ (*p < 0.05, **p < 0.01, ***p < 0.001; Mann Whitney U test). (B) All immunofluorescence microscopy experiments were performed on cultured cell after 3 days. DAPI(blue), Phalloidin(red) and Merged staining are shown in mono-cultured hTERT-hNOF(first panels) and hTERT-hNOF co cultured with HEK (second panels), YD10B OSCC cells (third panels) and YD38 OSCC cells (fourth panels), Scale bar, 50μm. The rectangular boxes are shown as enlarged sections to observe the F-actin assembly in left lower part.

To identify the cytoskeletal alteration of fibroblasts by co-culturing with OSCC cells, cell sizes were measured (region of interest, ROI). Compared with fibroblasts mono-cultured and co-cultured with HEK, the average of cell area significantly showed 1.42-and 1.27-fold increase in hTERT-hNOFs co-cultured with YD10B, and 1.52- and 1.35- fold increase in them with YD38, respectively (Fig 2A). These results indicated that fibroblasts showed larger cell size as stimulated by OSCC cells. Furthermore, primary CAFs, already stimulated by OSCC cells, have larger cell sizes than hTERT-hNOFs, respectively. When primary CAFs were additionally grown surrounded by OSCC cells (YD10B), the average of cell size is more increased than mono-cultured CAFs (CAF#1 with YD10B, 1.79-fold increase; CAF#2 with YD10B, 1.15-fold increase), like hTERT-hNOFs co-cultured with OSCC cells (Fig 2A). The results indicate that fibroblasts have enlarged by exposure to OSCC cells.

Cytoskeletal proteins, including phalloidin, indicate cellular morphology and intracellular stress fibers[30]. To further examine intracellular phenotypical alteration, phalloidin staining was performed. F-actin assembly was increased in the hTERT-hNOFs co-cultured with OSCC cells, but not in hTERT-hNOFs mono-cultured or co-cultured with HEK (Fig 2B).

### RhoA regulates cytoskeletal change of CAFs

**Fig 3:**
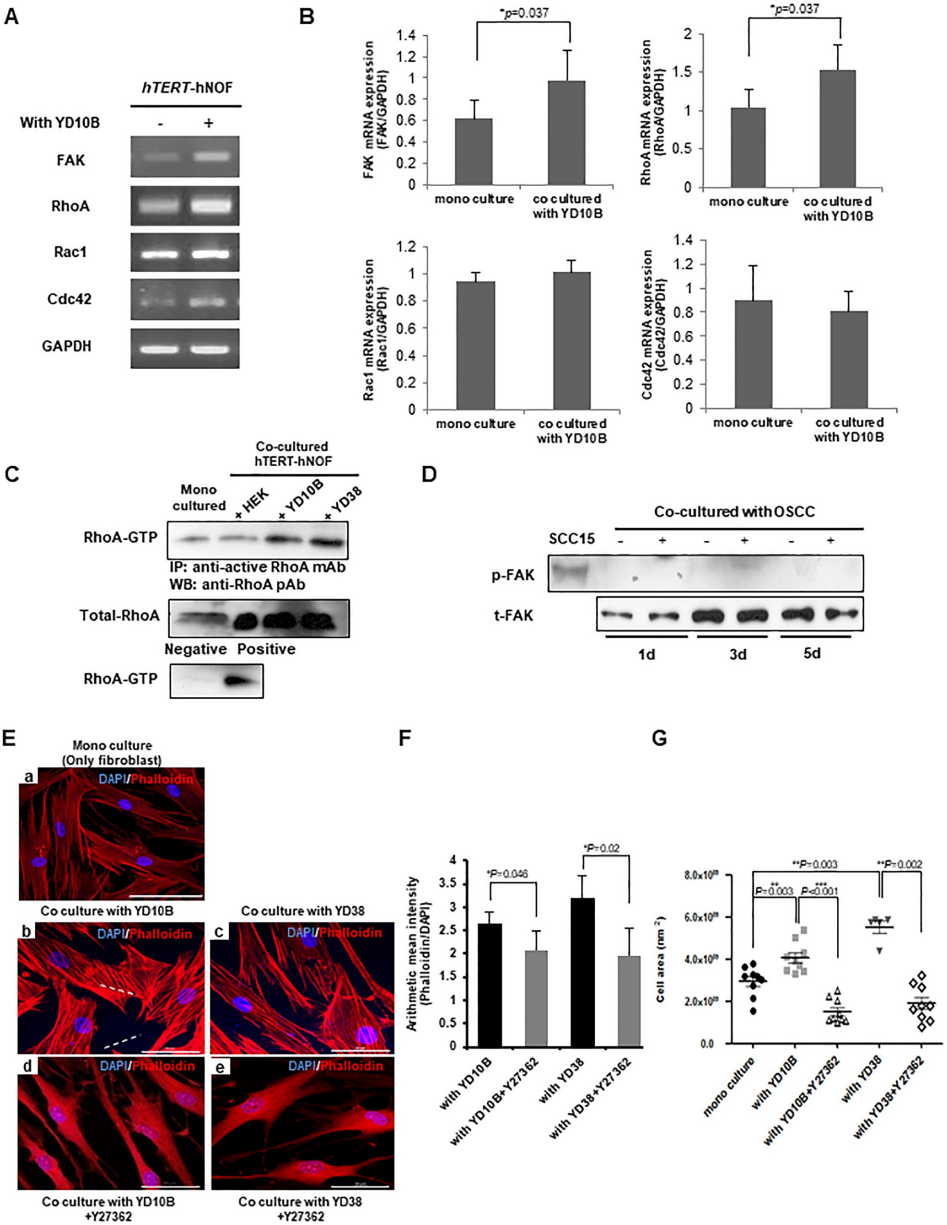
RhoA modulates cytoskeletal alteration in fibroblasts adjacent to OSCC cells. (A) hTERT-hNOFs were grown with YD10B OSCC cells for 24 h and then Rho GTPase family gene expression was identified by RT-PCR. (B) Quantification of Rho GTPase mRNA expression was shown. Each gene was normalized with ratio of GAPDH expression. The experiments were performed in three-independent (*p < 0.05; Mann-Whitney U test). (C) RhoA-GTP expression in hTERT-hNOFs mono-cultured and co-cultured with HEK or OSCC cells. Cell lysates were harvested at 24 h and then RhoA-GTP pull down assay was performed. (D) hTERT-hNOFs was grown with YD10B OSCC cells for indicated time. The phosphorylation of FAK(Tyr397) was identified by western blot. SCC 15 tongue SCC was used as a positive control of phospho-FAK. (E) All immunofluorescence microscopy experiments were performed on cultured cell after 3days. DAPI(blue), F-actin(Phalloidin)(red) and Merged staining were shown in mono-cultured hTERT-hNOF(left, top) and hTERT-hNOF co cultured with YD10B OSCC cell (left, middle), YD38 OSCC cell (right, middle), YD10B OSCC cell + Y27632(ROCK inhibitor, 10μg/ml) (left, bottom) and YD38 OSCC cell + Y27632(ROCK inhibitor, 10μg/ml) (right, bottom), Scale bar, 50μm. (F) The mean intensity of Phalloidin (F-actin) was shown. It was normalized by dividing the intensity of DAPI in cells (*p < 0.05; Mann-Whitney U test). (G) Cell size was measured by using ZEN 2012 software program. (mono cultured, n = 9; with YD10B, n = 9; with YD10B+Y27632, n = 9; with YD38, n = 5; with YD38+Y27632, n = 9) (*p < 0.05, **p < 0.01, ***p < 0.001; Mann-Whitney U test).

Rho GTPase family genes related to cytoskeletal changes of CAFs[31,32]. Among them, Focal adhesion kinase (FAK) and RhoA mRNA expression were markedly upregulated in hTERT-hNOFs co-cultured with YD10B cells (Fig 3A and 3B). Out of two Rho GTPase family genes, active RhoA protein expression was increased by co-culturing with OSCC cells, not with HEK (Fig 3C). However, no evidence of FAK phosphorylation was found (Fig 3D), suggesting that the cytoskeletal alteration of fibroblasts surrounding OSCC cells occurs independently on FAK phosphorylation.

Co-cultures were treated with the RhoA kinase (ROCK) inhibitor, Y27632, to assess whether RhoA regulates cytoskeletal alterations in hTERT-hNOFs co-cultured with OSCC cells. Y27632 induced the reduction of cell size and stress fibers (Fig 3E and 3G), suggesting that RhoA-ROCK signaling regulates the cytoskeletal change of CAFs.

### Nuclear localization of YAP increased in CAFs

**Fig 4:**
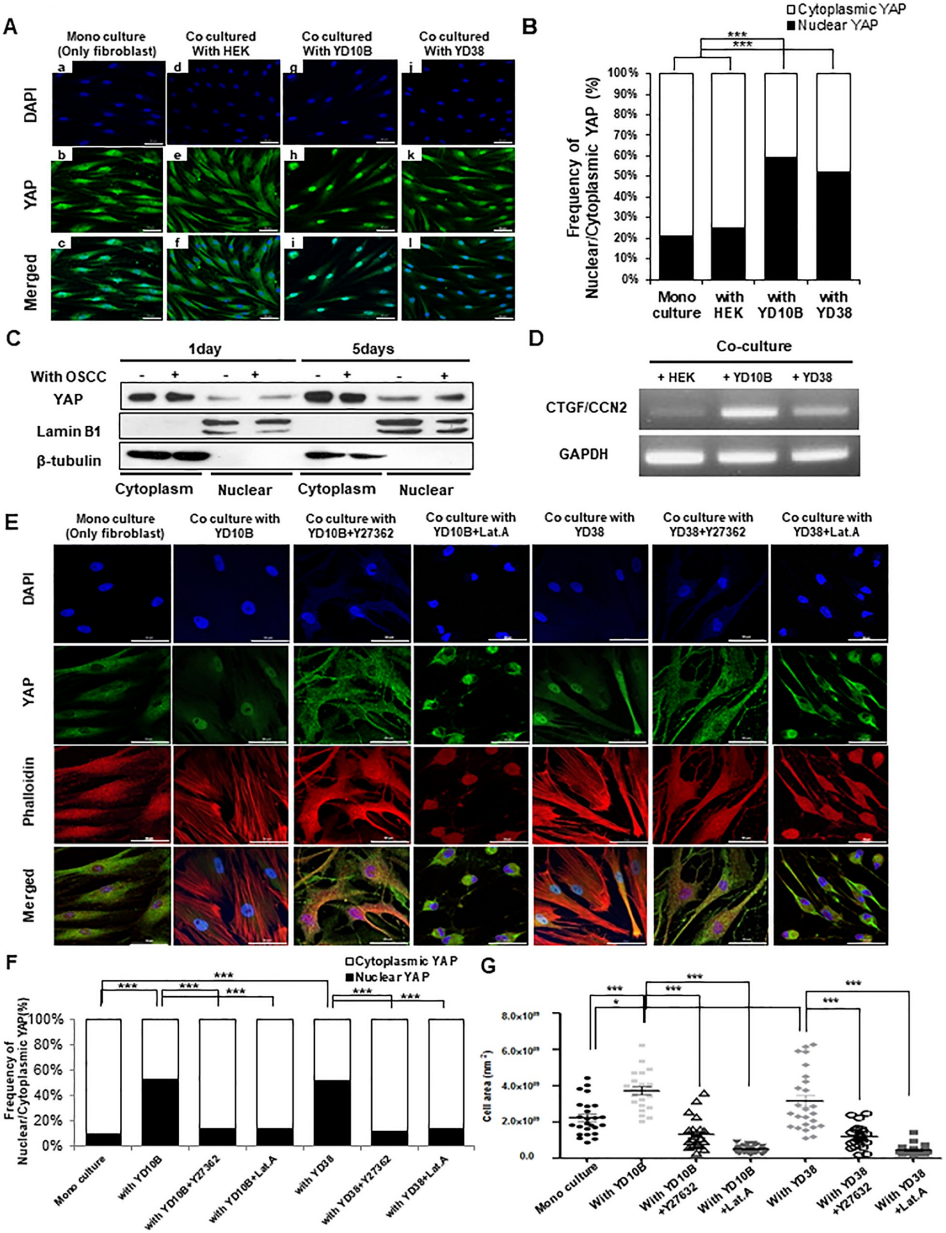
Nuclear accumulation of YAP was increased in CAFs. (A)All immunofluorescence microscopy experiments were performed on cultured cell after 3days. DAPI(blue), YAP(green) and Merged staining are shown in mono-cultured hTERT-hNOF(first panels) and hTERT-hNOF co cultured with HEK (second panels), YD10B OSCC cell (third panels) and YD38 OSCC cell (fourth panels), Scale bar, 50μm. (B) Graph summarizing the distribution of YAP was shown. It was analyzed by Image J software program (hTERT-hNOFs mono cultured, n = 67; co cultured with HEK, n = 53; with YD10B, n = 60; with YD38, n = 61, ***p< 0.001; student *t* test). (C) hTERT-hNOFs were grown with or without YD10B OSCC cell for indicated time. Nuclear/cytoplasmic fraction was isolated from each protein and then western blot was performed to check the YAP distribution. Lamin B1 was used as a positive control of nuclear protein fraction and β-tubulin was used as a positive control of cytoplasmic protein fraction. Nuclear/cytoplasmic YAP expression was normalized to each nuclear/cytoplasmic positive control. (D) hTERT-hNOFs were grown with OSCC cells or HEK cell for 3 days. YAP target gene; CTGF, was identified by RT-PCR. (E)All immunofluorescence microscopy experiments were performed on cultured cell after 3days. DAPI(blue), Phalloidin(red) YAP(green) and Merged staining are shown in mono-cultured hTERT-hNOF(first line) and hTERT-hNOF co cultured with YD10B OSCC cell (second line), with YD10B OSCC cell + Y27362 (third line), with YD10B OSCC cell + Lat.A (fourth line), with YD38 OSCC cell (fifth line), with YD38 OSCC cell +Y27362 (six line) and with YD38 OSCC cell + Lat.A (seven line), Scale bar, 50μm. (F) Graph summarizing the distribution of YAP was shown. It was analyzed by Image J software program (mono culture, n = 26; with YD10B, n = 13; with YD10B+Y27362, n = 35; with YD10B+Lat.A, n = 38; with YD38, n = 24; with YD38+Y27632, n = 34; with YD38+Lat.A, n = 39) (***p < 0.001; student *t* test). (G) Cell size was measured by using ZEN 2012 software program. (mono culture, n = 25; with YD10B, n = 22; with YD10B+Y27362, n = 24; with YD10B+Lat.A, n = 25; with YD38, n = 26; with YD38+Y27632, n = 24; with YD38+Lat.A, n = 25) (*p < 0.05, **p < 0.01, ***p < 0.001; student *t* test).

Considering that YAP acts as a downstream of RhoA[30,33], YAP localization was identified in co-cultured hTERT-hNOFs with OSCC cells by immunofluorescence. When hTERT-hNOFs were grown alone or with HEK, YAP was mainly accumulated in cytoplasm. Conversely, YAP was mainly localized in the nucleus of the fibroblasts as co-cultured with OSCC cells (Fig 4A and 4B). To confirm these results, nuclear and cytoplasmic protein was separately extracted. Following co-culture hTERT-hNOFs with OSCC cells, the cytoplasmic expression of YAP increased 1.4-fold, whereas its nuclear expression increased 2.8-fold compared to mono-cultured cells (Fig 4C). Consistent with these results, connective tissue growth factor (CTGF), a YAP target gene, was increased in hTERT-hNOFs co-cultured with OSCC cells, compared to co-culturing with HEK cells (Fig 4D).

To further elucidate the relationship between RhoA and YAP localization or cytoskeleton and YAP localization, ROCK inhibitor (Y27362) or actin polymerization inhibitor (Lat.A) was treated. After the treatment of Y27632 or Lat.A, nuclear YAP was relocalized at cytoplasm in co-cultured hTERT-hNOF with OSCC cells (Fig 4E and 4F). Also, the cell size was reduced by the treatment of Y27362 or Lat.A (Fig 4E and 4G). To support these results, phalloidin (F-actin) expression and YAP distribution was checked by immunofluorescence in NOF and CAFs (S3 Fig). As results, YAP was mostly distributed at cytoplasm in NOF, whereas YAP was localized at the nucleus in CAFs with distinct stress fibers (S3A and S3B Fig). The cell size of CAFs was larger than that of NOF, even this result was not statistically significant (S3C Fig).

### YAPS127A fibroblasts increase migratory and invasive activities of OSCC cells by remodeling ECM structures

**Fig 5:**
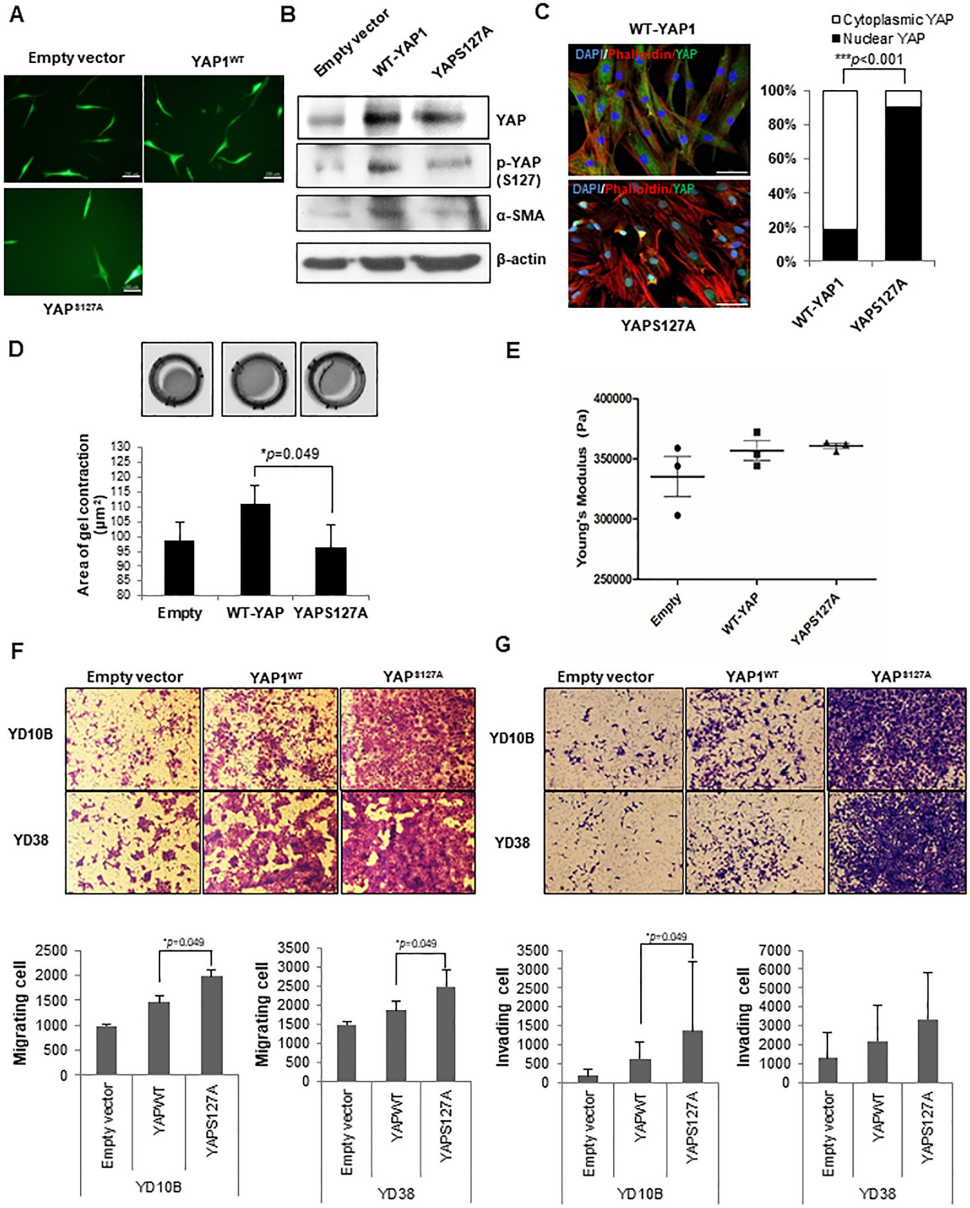
YAPS127A fibroblasts rearrange ECM structures and promote motility and invasiveness of OSCC cells. (A) Fluorescence microscope was used to check the expression of EGFP on cultured cell after transfection with pcDNA4-HisMaxB-YAP1 and YAPS127A, Scale bar, 200μm. (B) The YAP, p-YAP(S127) and αSMA protein expression was identified in all transfected cells (empty-, WT-YAP-, YAPS127A transfected hTERT-hNOFs) by Western blot. (C) Immunofluorescence images were shown in WT-YAP1- and YAPS127A-hTERT-hNOFs. Merged stained Images (DAPI(blue), F-actin(Phalloidin)(red), YAP(green))are shown. Scale bar, 50μm. The bar graph indicates the distribution of YAP in WT-YAP1- and YAPS127A-hTERT-hNOFs. It was analyzed by Image J software program (WT-YAP1-hTERT-hNOFs, n = 40; YAPS127A-hTERT-hNOFs, n = 40) (***p < 0.001; Student *t* test). (D) The representative images show gel contractility by the fibroblasts. Histogram shows mean value ± SD (n = number of gels (Empty, 3; WT-YAP, 3; YAPS127A, 3), assessed over multiple experiments (*p < 0.05; Mann Whitney U test). (E) Histogram shows elastic modulus of matrix remodeled by fibroblasts, respectively. Each data point represents a single measurement, (n = 3, respectively) measurements in total. Line and error bars indicate mean value ± SD. (F-G) Representative images showed the (F) migration and (G) invasion of OSCC cells (YD10B and YD38) cultured with each of fibroblasts, which were transfected with Empty-, WT-YAP-, YAPS127A-transfected hTERT-hNOFs). Quantification of OSCC cells (YD10B and YD38) migration toward lower chamber is shown as bar graphs. Each assay was performed in independent triplicate repeats (*p < 0.05; Mann-Whitney U test).

EGFP was co-transfected with pcDNA4-HisMaxB(empty vector), pcDNA4-HisMaxB-YAP1(WT) and pcDNA4-HisMaxB-YAPS127A in hTERT-hNOFs. Fluorescence microscope was used to identify the transfection of EGFP (Fig 5A). Furthermore, YAP protein expression was identified by western blot. YAPS127A-fibroblasts showed lower expression phospho-YAP(S127), which indicates cytoplasmic retention, compared with WT fibroblasts (Fig 5B). Immunofluorescent features revealed YAP nuclear localization in YAPS127A-fibroblasts, compared with WT-fibroblasts (Fig 5C). YAPS127A fibroblasts showed more enlarged cell size than WT-fibroblasts (S4B Fig). However, the intensity of phalloidin was not significant (S4A Fig). To test whether YAPS127A fibroblasts can regulate the contractility of gel, the contractility of collagen gels was measured by using their matrix remodeling capacity. Significantly, the gel of YAPS127A fibroblasts was contracted, compare with the gel of WT fibroblasts (Fig 5D), indicating that YAPS127A fibroblasts can remodel the matrix. Furthermore, the matrix stiffness showed that 1.08- /1.01- fold increase in YAPS127A fibroblasts (360633.3 Pa), respectively, compared with in empty vector fibroblast (335366.7 Pa) or WT-fibroblast (356866.7 Pa) (Fig 5E).

**Fig 6:**
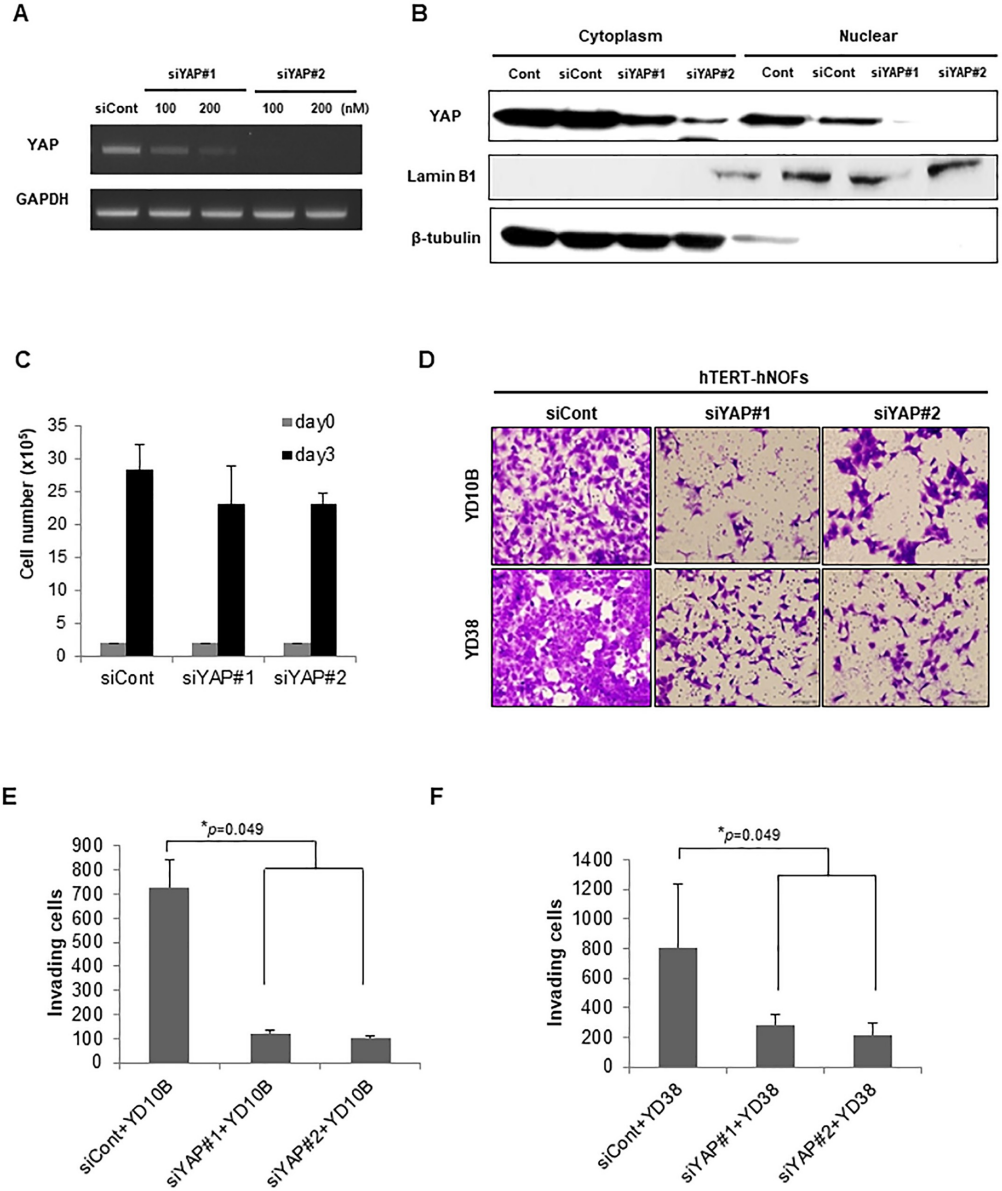
YAP-silencing fibroblasts attenuate invasiveness in OSCC cells. (A) The depletion of YAP with 2 different siRNAs was identified by RT-PCR. Representative image is shown. (B) Nuclear/cytoplasmic fraction was isolated from each protein and then western blot was performed to check nuclear/cytoplasmic YAP expression by siRNAs targeting YAP. Lamin B1 was used as a positive control of nuclear protein fraction and β-tubulin was used as a positive control of cytoplasmic protein fraction. (C) Cell proliferation in siCont- and siYAPs-transfected hTERT-hNOFs. Cells (2 x 10^5^) were seeded into well and then counted after 3 days. (D) Representative images of invading OSCC cells toward lower chamber are shown (magnification: 200X, scale bar: 100 μm). (E, F) The bar graphs indicated a quantification of invading YD10B (E) or YD38 (F) OSCC cells as siYAPs-transfected hTERT-hNOFs were placed in the lower chamber by comparison with siCont transfected hTERT-hNOFs. This assay (E and F) was performed in independent triplicate repeats (*p < 0.05; Mann-Whitney U test).

To further evaluate whether RhoA-YAP induced cytoskeletal changes contribute to both migratory and invasive activities of OSCC cells, transwell migration and invasion assays were carried out after co-cultivation with each transfected-fibroblasts. YAPS127A fibroblasts induced higher cell motility of OSCC than WT-YAP fibroblasts or empty vector-fibroblasts (Fig 5F). Similarly, YAPS127A fibroblasts induced higher invasive activity than the wild type or the control cells (Fig 5G). To ascertain that YAP plays a critical role in OSCC invasiveness, two types of siRNA targeting YAP were used (Fig 6A). Subsequently, the effects of siRNAs on YAP nuclear localization were observed by using 200nM siYAPs. Both types of siRNA targeting YAP dramatically reduced nuclear YAP, whereas cytoplasmic YAP was still remained (Fig 6B). To examine the effect of matrix remodeling on gels by silencing YAPs fibroblasts, we performed contraction assay and roughness test of gels. As results, siYAPs-transfected fibroblasts showed less contraction and less rough surface of gels than siCont-transfected fibroblasts, even though it is not statistically significant (S5A and S5B Figs), making it permissive to OSCC cell invasion. Consequently, knockdown of YAP significantly reduced the number of invading OSCC cells that it shows to 6.00- / 7.02- fold decrease in YD10B cells and to 2.83- / 3.75- fold decrease in YD38 cells, respectively, compare with OSCC cells grown with siCont-transfected fibroblasts (Fig 6D, 6E and 6F). These results are independent on reduced cell proliferation showing that knockdown of YAP showed 1.23- fold and 1.22- fold decreased cell proliferation, compared with siCont-transfected fibroblasts, which data were not significant (Fig 6C).

## Discussion

For this study, immortalized human normal oral fibroblasts (hTERT-hNOFs) were used to obtain repeatable *in vitro* results. Following co-culture with OSCC cells, the hTERT-hNOFs showed biologically identical characteristics with CAFs, and the functionally enhanced invasiveness of OSCC cells. These results suggest that hTERT-hNOFs can substitute for CAFs by co-culture with OSCC cells. In spite of hTERT-overexpression in fibroblasts, the fibroblasts showed a senescent phenotype after co-culture with OSCC cells. It might be triggered by stress from stimuli derived from OSCC cells. Stress-induced senescence cannot be prevented by hTERT-mediated telomere elongation and is triggered not only by shortening telomere, but also by nonspecific genome-wide DNA damage[34,35].

RhoA-ROCK, which could induce stress fiber formation with Rho GTPase family, is implicated in CAF-mediated remodeling of tumor microenvironment[25,26,31]. In our current study, we verified that RhoA mRNA and protein expression increased in hTERT-hNOFs by co-culturing with OSCC cells. Enlarged cell size and formation of stress fibers were interrupted by RhoA-ROCK inhibitor, supporting that RhoA-ROCK signaling could contribute to cytoskeletal alteration in CAFs.

YAP activity is dependent on its distribution; nuclear localization induces several growth factors via binding to different transcription factors, such as p73, RUNX2 and TEADs[32,36]. Conversely, cytoplasmic localization indicates the inactivated form, due to Ser127 phosphorylation, leading to its cytoplasmic retention[32,33]. In our study, YAP was mainly localized in the nucleus and the expression of CTGF, direct target gene of YAP and TEADs, was increased in co-cultured hTERT-hNOFs with OSCC cells. In addition, nuclear distribution of YAP was translocalized to the cytoplasm by inhibitors (Y27632 and Lat.A) in co-cultured hTERT-hNOFs with OSCC cells, confirming that YAP distribution can be regulated by RhoA-ROCK signaling and cytoskeleton organization.

To search the role of nuclear YAP-localized fibroblasts in cancer progression, a YAP active-mutant (YAPS127A) was generated. As a result, the gel contractility and stiffness increased with enlarged cellular size in YAPS127A fibroblasts. These results correspond to the reports that nuclear YAP as well as RhoA-ROCK affect cytoskeletal reorganization via RhoA-YAP circular loops[33,37].

Regarding that the increased gel contractility and tissue stiffness may act as significant tumor promoters[38], we observed migratory and invasive capacity, resulting in enhanced invasiveness of OSCC cells when co-cultured with YAPS127A fibroblasts. Matrix stiffness accompanies epithelial malignancy with tissue fibrosis and promotes tumor invasion by enhancing integrin-dependent mechanotransduction[39]. Given the data that YAP of fibroblasts play a critical role in invasiveness through modulating matrix structure[40,41], we examined the invasiveness by YAP-silencing in fibroblasts, revealing the reduced invasiveness in OSCC cells along with ECM rearrangement. Taken together, we can extrapolate that remodeling matrix structure by YAP-silencing may be a novel promising target for cancer therapy. Currently, CAFs-targeting cancer therapy has been limited to inhibit signaling molecules expressed in CAFs or cancer cells-CAFs interaction[19,42]. In the near future, the novel trial targeting matrix remodeling can be emerged.

In summary, we demonstrated that CAFs showed cytoskeletal alteration *via* RhoA-ROCK and increased nuclear YAP localization. Consequently, YAP activity of fibroblasts could remodel matrix organization and increase the matrix stiffness, in turn, promote the migration and invasion of OSCC cells. Thus, targeting cytoskeletal alterations in CAFs might be a spotlight in novel therapeutic approaches designed to modulate mechanical behaviors of CAF that contribute to OSCC invasiveness.

## Supporting information

S1 Fig Comparison of p16 and Ki67 expression in hTERT-hNOFs between co-cultured with HEK and with OSCC cells. All immunofluorescence representative pictures were shown on hTERT-hNOFs co-cultured with HEK (Upper panels), YD10B OSCC cells (Middle panels) and YD38 OSCC cells (Lower panels) for 3 days. DAPI(blue), Ki67(green) and p16(red) was stained, Scale bar, 50μm. Each bar graph indicates that Ki67 positive cells are represent as green, dual positivity of Ki67 and p16 as purple and p16 positivity as red for quantification. The number of positive cells was normalized by dividing the number of total cells, respectively, and presented as % of Ki67, Ki67+p16 and p16 positive cells. The results are shown as mean value ± SD (n = 3).
(TIF)

S2 Fig hTERT-hNOFs have characteristics of senescent phenotype adjacent to oral cancer cells. (A) SA-β-Gal assay was performed and representative microscopic pictures (a) mono-cultured hTERT-hNOFs, hTERT-hNOFs, co-cultured with OSCC cells, including (b) YD10B, (c) YD38, (d) YD9 and (e) HSC2 are shown at 72 h (magnification: 200X, scale bar: 500 μm). The quantification of SA-β-Gal positive cells was normalized by dividing the number of total cells and presented as % of SA-β-Gal positive cells. The results are shown as mean value ± SD (n = 3) (*p < 0.05; Mann Whitney U test). (B) Representative microscopic pictures of SA-β-Gal positive cells in (a) mono-cultured hTERT-hNOFs, (b) hTERT-hNOFs co-cultured with HEK, (c) hTERT-hNOFs co-cultured with OSCC YD10B cells and (d) YD38 cells at 72 h (magnification: 200X, scale bar: 500 μm). Enlarged images are shown in the bottom of each left panel. The number of SA-β-Gal positive cells was normalized by dividing the number of total cells and presented as % of SA-β-Gal positive cells. The results are shown as mean value ± SD (n = 3) (*p < 0.05; Mann Whitney U test).
(TIF)

S3 Fig F-actin assembly and YAP nuclear localization between NOF and CAFs. (A) All immunofluorescence microscopy experiments were performed on cultured cell after 3days. DAPI(blue), Phalloidin(red) and Merged staining are shown in mono-cultured hTERT-hNOF(first panels) and hTERT-hNOF co cultured with HEK (second panels), YD10B OSCC cell (third panels) and YD38 OSCC cell (fourth panels), Scale bar, 50μm. The rectangular boxes are shown enlarged section to observe the YAP localization and F-actin assembly in detail. (B) The bar graph indicates the distribution of YAP in NOF and CAFs. It was analyzed by Image J software program (NOF, n = 9; CAFs, n = 23(CAF#1, n = 7; CAF#2, n = 7; CAF#3, n = 11)). (C) Cell size was measured by using ZEN 2012 software program (NOF, n = 9; CAFs, n = 23(CAF#1, n = 7; CAF#2, n = 7; CAF#3, n = 11)) (*p < 0.05, ** *p* < 0.01, *** *p* < 0.001; Mann Whitney U test).
(TIF)

S4 Fig The intensity of F-actin and cellular size in WT- and YAPS127A fibroblasts. **(**A) The mean intensity of Phalloidin was shown in WT- and YAPS127A fibroblasts. It was normalized by dividing the intensity of DAPI in cells. (B) Cell size was measured by using ZEN 2012 software program (WT-fibroblasts and YAPS127A fibroblasts, n = 15, respectively) (*p < 0.05; Mann Whitney U test).
(TIF)

S5 Fig The rearrangement of ECM in siCont- and siYAPs fibroblasts. **(**A) The representative gel-contracting images were shown in siCont- and siYAPs fibroblasts. The bar graphs indicated average of gel size after contracting (B) The surface roughness (nm) by siCont- and siYAPs fibroblasts. it was measured by Atomic force microscopy(AFM). The experiments were performed in triplicate.
(TIF)

S1 Materials and Methods (DOCX)
