# Supplementary figures and images for: Cytoskeletal alteration modulates cancer cell invasion through RhoA-YAP signaling in stromal fibroblasts
Source: PLoS One. 2019 Mar 28;14(3):e0214553. doi: 10.1371/journal.pone.0214553 (PMC6438594; doi:10.1371/journal.pone.0214553)

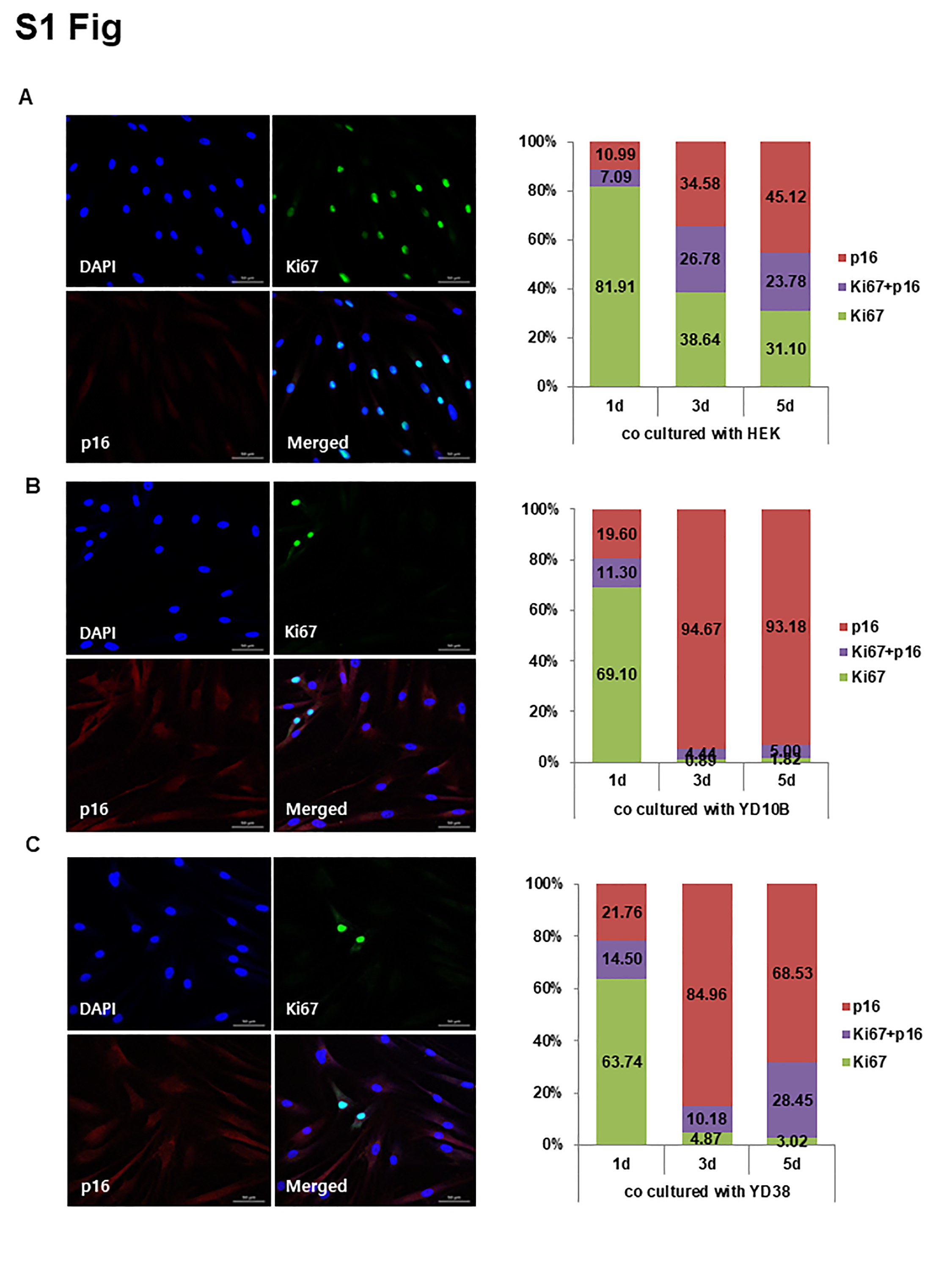

Supplement: S1 Fig — All immunofluorescence representative pictures were shown on hTERT-hNOFs co-cultured with HEK (Upper panels), YD10B OSCC cells (Middle panels) and YD38 OSCC cells (Lower panels) for 3 days. DAPI(blue), Ki67(green) and p16(red) was stained, Scale bar, 50μm. Each bar graph indicates that Ki67 positive cells are represent as green, dual positivity of Ki67 and p16 as purple and p16 positivity as red for quantification. The number of positive cells was normalized by dividing the number of total cells, respectively, and presented as % of Ki67, Ki67+p16 and p16 positive cells. The results are shown as mean value ± SD (n = 3). (TIF) [file pone.0214553.s001.TIF]

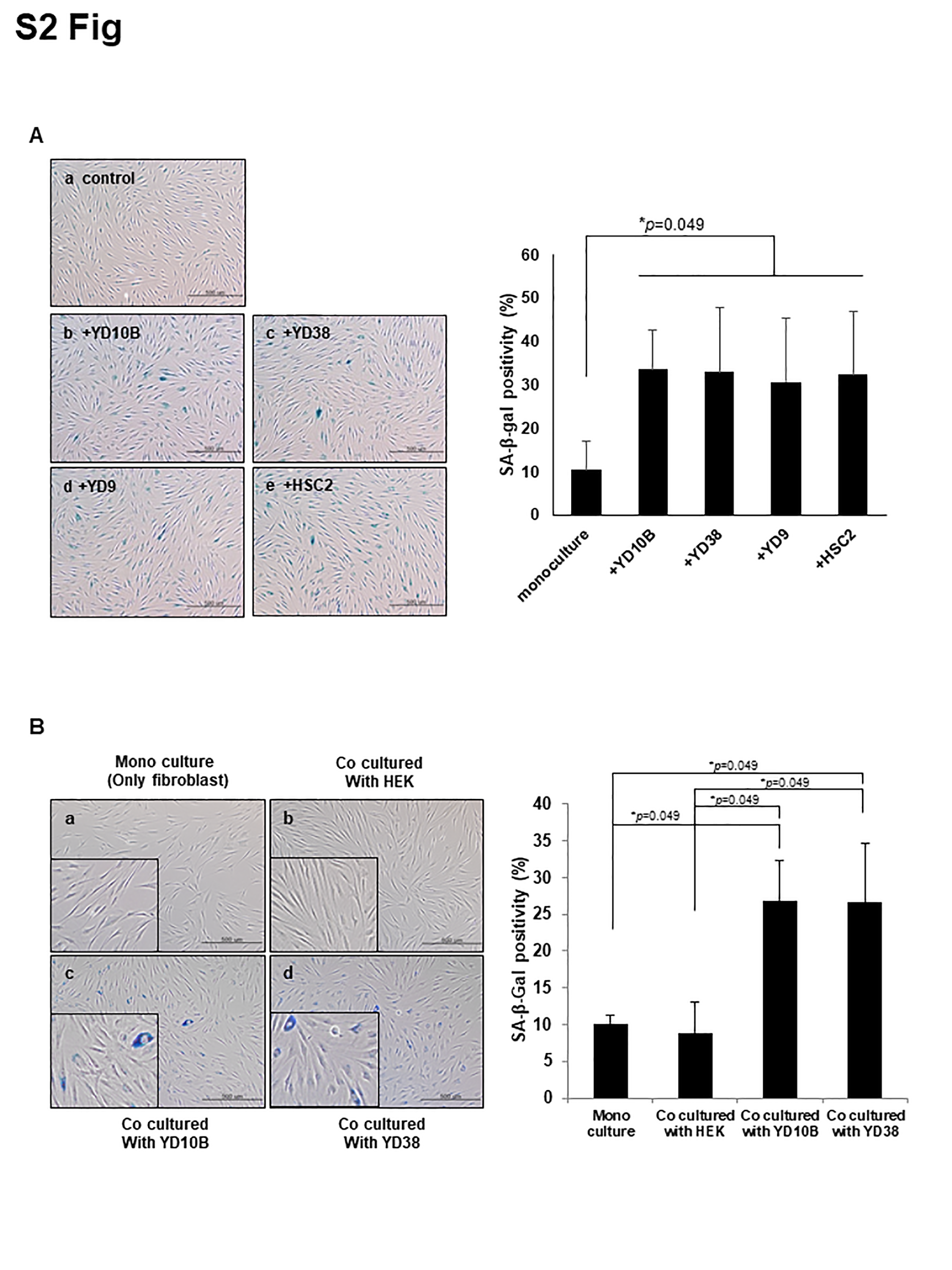

Supplement: S2 Fig — (A) SA-β-Gal assay was performed and representative microscopic pictures (a) mono-cultured hTERT-hNOFs, hTERT-hNOFs, co-cultured with OSCC cells, including (b) YD10B, (c) YD38, (d) YD9 and (e) HSC2 are shown at 72 h (magnification: 200X, scale bar: 500 μm). The quantification of SA-β-Gal positive cells was normalized by dividing the number of total cells and presented as % of SA-β-Gal positive cells. The results are shown as mean value ± SD (n = 3) (*p < 0.05; Mann Whitney U test). (B) Representative microscopic pictures of SA-β-Gal positive cells in (a) mono-cultured hTERT-hNOFs, (b) hTERT-hNOFs co-cultured with HEK, (c) hTERT-hNOFs co-cultured with OSCC YD10B cells and (d) YD38 cells at 72 h (magnification: 200X, scale bar: 500 μm). Enlarged images are shown in the bottom of each left panel. The number of SA-β-Gal positive cells was normalized by dividing the number of total cells and presented as % of SA-β-Gal positive cells. The results are shown as mean value ± SD (n = 3) (*p < 0.05; Mann Whitney U test). (TIF) [file pone.0214553.s002.TIF]

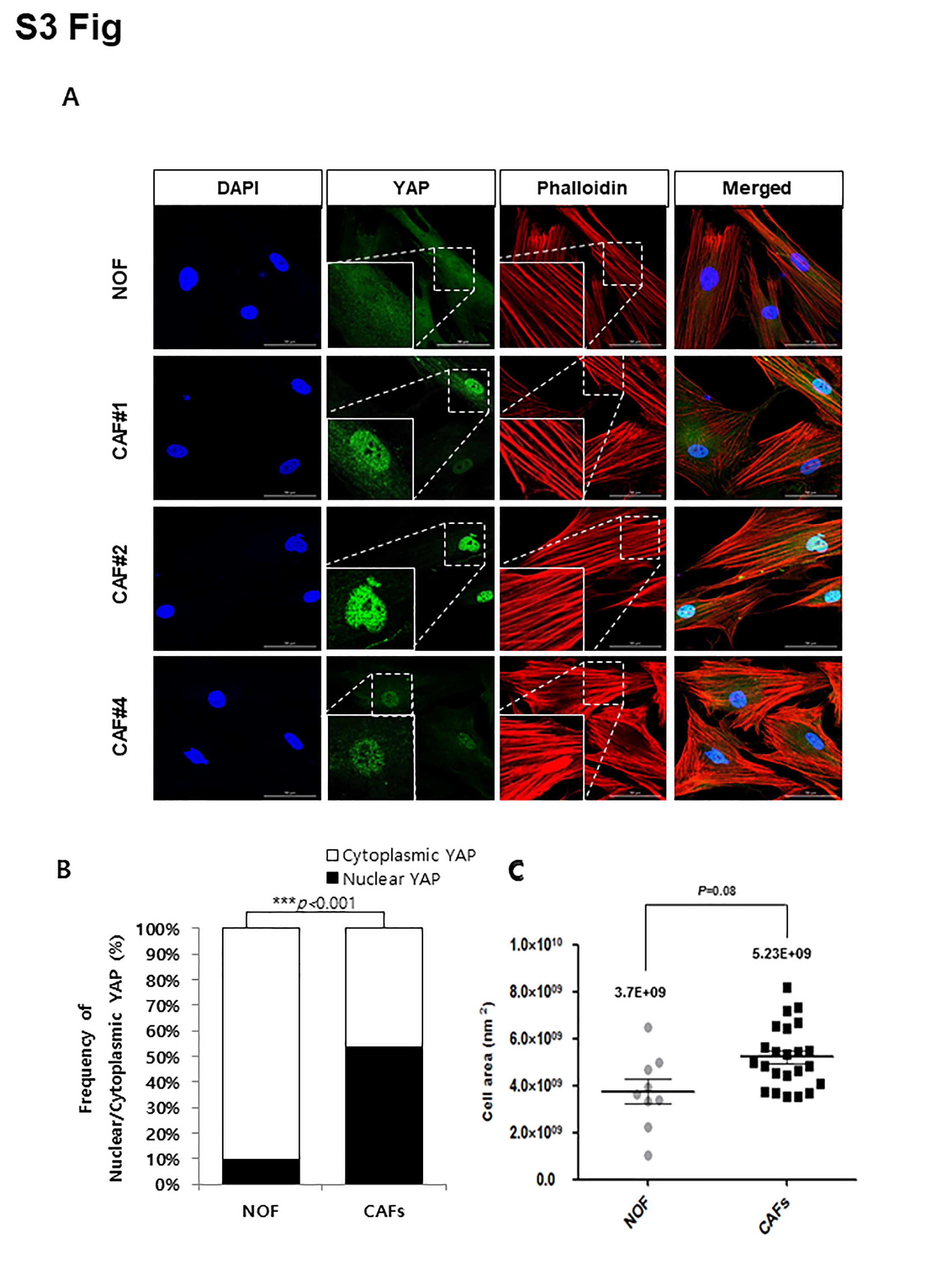

Supplement: S3 Fig — (A) All immunofluorescence microscopy experiments were performed on cultured cell after 3days. DAPI(blue), Phalloidin(red) and Merged staining are shown in mono-cultured hTERT-hNOF(first panels) and hTERT-hNOF co cultured with HEK (second panels), YD10B OSCC cell (third panels) and YD38 OSCC cell (fourth panels), Scale bar, 50μm. The rectangular boxes are shown enlarged section to observe the YAP localization and F-actin assembly in detail. (B) The bar graph indicates the distribution of YAP in NOF and CAFs. It was analyzed by Image J software program (NOF, n = 9; CAFs, n = 23(CAF#1, n = 7; CAF#2, n = 7; CAF#3, n = 11)). (C) Cell size was measured by using ZEN 2012 software program (NOF, n = 9; CAFs, n = 23(CAF#1, n = 7; CAF#2, n = 7; CAF#3, n = 11)) (*p < 0.05, ** p < 0.01, *** p < 0.001; Mann Whitney U test). (TIF) [file pone.0214553.s003.TIF]

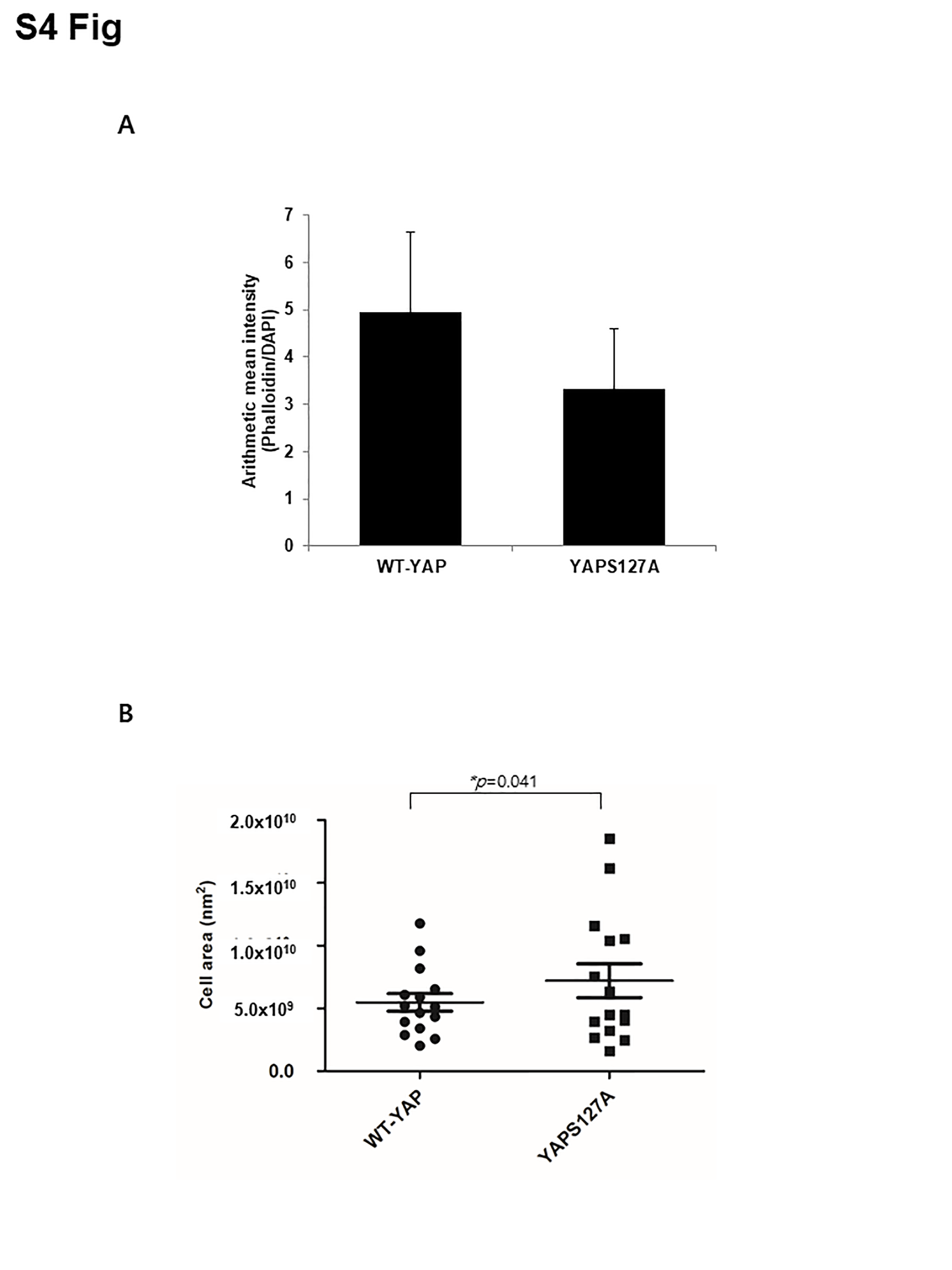

Supplement: S4 Fig — (A) The mean intensity of Phalloidin was shown in WT- and YAPS127A fibroblasts. It was normalized by dividing the intensity of DAPI in cells. (B) Cell size was measured by using ZEN 2012 software program (WT-fibroblasts and YAPS127A fibroblasts, n = 15, respectively) (*p < 0.05; Mann Whitney U test). (TIF) [file pone.0214553.s004.TIF]

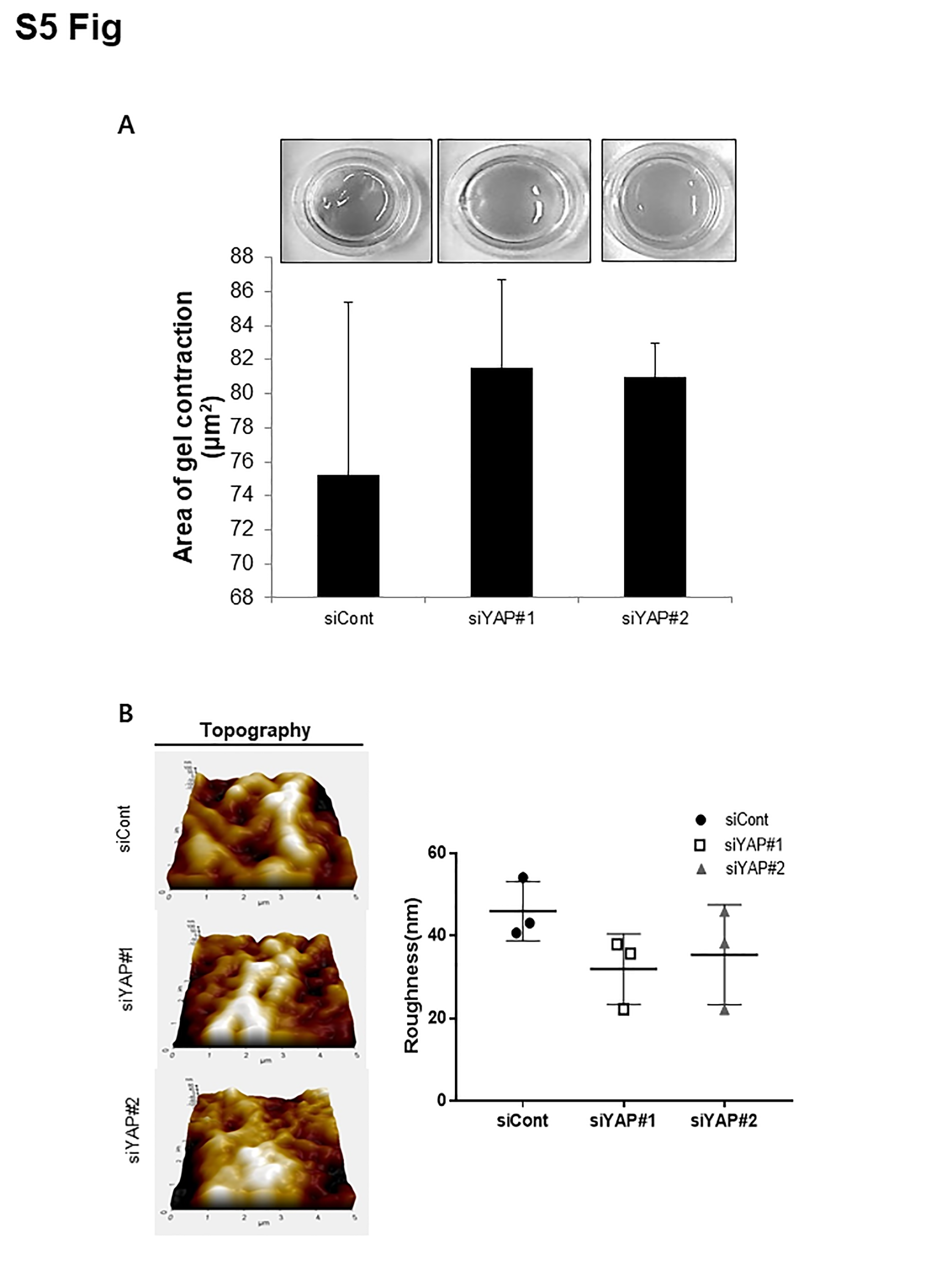

Supplement: S5 Fig — (A) The representative gel-contracting images were shown in siCont- and siYAPs fibroblasts. The bar graphs indicated average of gel size after contracting (B) The surface roughness (nm) by siCont- and siYAPs fibroblasts. it was measured by Atomic force microscopy(AFM). The experiments were performed in triplicate. (TIF) [file pone.0214553.s005.TIF]
