# Supplementary material for: Cytoskeletal alteration modulates cancer cell invasion through RhoA-YAP signaling in stromal fibroblasts
Source: PLoS One. 2019 Mar 28;14(3):e0214553. doi: 10.1371/journal.pone.0214553 (PMC6438594; doi:10.1371/journal.pone.0214553)
Supplement: S1 Materials and Methods — (DOCX) [file pone.0214553.s006.docx]

***Supplementary data for***

**Cytoskeletal alteration modulates cancer cell invasion through RhoA-YAP signaling in stromal fibroblasts**

Do Kyeong Kim^1,2^, Eun Kyoung Kim^1^, Da-Woon Jung^3^ and Jin Kim^1^ ^*^

**Authors’ Affiliations:** ^1^ Oral Cancer Research Institute, Department of Oral Pathology, BK21 PLUS Project, Yonsei University College of Dentistry, Seoul 03722, Republic of Korea ^2^Department of Dental Hygiene, Jeonju Kijeon College, Jeonju, 54989, Republic of Korea ^3^ New Drug Targets Laboratory, School of Life Sciences, Gwangju Institute of Science and Technology, 1 Oryong-dong, Gwangju 500-712, Republic of Korea

**Corresponding Author:** Jin Kim, Oral Cancer Research Institute, Department of Oral Pathology, Yonsei University College of Dentistry, Seoul 03722, Korea. Phone: +82-2-2228-3031; Fax: +82-2-392-2959; E-mail: jink@yuhs.ac

**Supplementary Materials and Methods**

**Cell culture**

All fibroblasts (hTERT-hNOFs, CAFs and NOFs) were then maintained in F medium which is composed of Dulbecco’s modified Eagles medium (Gibco BRL, NY, USA) and F-12 Ham (Ham’s F12; Gibco BRL, NY, USA) mixed in a 3:1 ratio, and supplemented with 10% fetal bovine serum and 1% penicillin/streptomycin. Normal human epidermal keratinocytes (HEK) maintained in keratinocyte growth media with supplementary bullet kit (KGM; Lonza, Walkersville, MD, USA). HSC2 OSCC cells were grown in F medium and YD OSCC cells were maintained in EF medium (the mixture of F medium and E medium at 9: 1 ratio). YD-9 were from buccal check of OSCC patients[1]. All cells were maintained at 37 °C in an incubator containing 5% CO_2_. The authentication of all cell lines was verified by STR DNA profiling in Korean Cell Line Bank.

**Reagents and antibodies**

For immunofluorescence, Phalloidin-Tetramethylrhodamine B isothiocyanate and diamidinophenylindole (DAPI) were purchased from Sigma Aldrich (MO, USA). Anti-Ki67 (1:50, Rabbit monoclonal, Cell signaling technology, Beverly, MA, USA) and Anti-p16 (1:100, mouse monoclonal, Santa Cruz, CA, USA) was used. The cells cover-slipped with Dako fluorescent mounting medium (Dako, CA, USA)

**Senescence-associated β-galactosidase staining**

In co-culturing condition, hTERT-hNOFs (1.5 × 10^5^) were seeded and incubated overnight in a 6-well lower chamber. A variety of OSCC cells, including YD10B, YD38, YD9, YD32 and HSC2 (1.5 × 10^5^) were added in 6-well upper chamber, respectively. In detail, the methods of senescence-associated β-galactosidase staining were described in original materials and methods

**Immunofluorescence**

The cells were stained with appreciated antibodies (p16, Ki67, Phallodin(F-actin) and YAP) following manufacturer’s instructions. In detail, the methods were described in original materials and methods.

**Gel contraction assay**

To assess the matrix remodeling mediated by fibroblasts, respectively, the siCont- and siYAPs fibroblasts were embedded in 500 μl of collagen Type I and seeded into. After 5 days, the gel contraction value was obtained and the relative diameters of the well and the gel were measured using Image J software. In detail, the methods were described in original materials and methods.

**Topography**

To check the surface roughness, the fibroblasts were grown on cover glass with collagen type I. After 3 days, the experiment samples were measured by using Atomic force microscopy(AFM) in Yonsei center for Research Facilities.

**Statistical analysis**

All statistical analyses were analyzed by using the SPSS version 20 (SPSS Inc., Chicago, IL, USA). Mann-Whitney *U* test and student *t*-test were used to determine the statistical significant difference. All of the variables were tested in three independent experiments, and each experiment was performed at least in triplicate. The results are shown as the mean ± standard deviation (SD). The value of **p* < 0.05, ***p* < 0.01 and ****p* < 0.001 was considered statistically significant.

**References**

1. Lee EJ, Kim J, Lee SA, Kim EJ, Chun YC, Ryu MH, et al. Characterization of newly established oral cancer cell lines derived from six squamous cell carcinoma and two mucoepidermoid carcinoma cells. Exp Mol Med. 2005;37: 379-390. doi:10.1038/emm.2005.48 PMID:16264262
